# Number of days required to estimate physical activity constructs objectively measured in different age groups: Findings from three Brazilian (Pelotas) population-based birth cohorts

**DOI:** 10.1371/journal.pone.0216017

**Published:** 2020-01-10

**Authors:** Luiza Isnardi Cardoso Ricardo, Andrea Wendt, Leony Morgana Galliano, Werner de Andrade Muller, Gloria Izabel Niño Cruz, Fernando Wehrmeister, Soren Brage, Ulf Ekelund, Inácio Crochemore M. Silva

**Affiliations:** 1 Postgraduate Program in Epidemiology, Federal University of Pelotas, Pelotas, Brazil; 2 Postgraduate Program in Physical Education, Federal University of Pelotas, Pelotas, Brazil; 3 MRC Epidemiology Unit, University of Cambridge, Cambridge, England, United Kingdom; 4 Department of Sport Medicine, Norwegian School of Sport Sciences, Oslo, Norway; Indiana University-Purdue University Indianapolis, UNITED STATES

## Abstract

**Purpose:**

The present study aims to estimate the minimum number of accelerometer measurement days needed to estimate habitual physical activity (PA) among 6- (2010), 18- (2011) and 30- (2012) year-old participants, belonging to three population-based Brazilian birth cohorts.

**Method:**

PA was assessed by triaxial wrist-worn GENEActiv accelerometers and the present analysis is restricted to participants with at least 6 consecutive days of measurement. Accelerometer raw data were analyzed with R-package GGIR. Description of PA measures (overall PA, moderate-to-vigorous PA (MVPA), light PA (LPA)) on weekdays and weekend days were conducted, and statistical differences were tested with chi-squared and Kruskal-Wallis tests. Spearman Brown Formulae was applied to test reliability of different number of days of accelerometer use.

**Results:**

Differences between week and weekend days regarding LPA, MVPA and overall PA, were only observed among 30-year-olds. Higher levels of MVPA (p = 0.006) and overall PA (p<0.001) were identified on weekdays. For overall PA, to achieve a reliability coefficient >0.70, two and three days of measurement were needed in adults and children, respectively. For LPA, a reliability coefficient >0.70 was achieved with five days in 6-year-old children, three days in 18-year-old young adults, and four days in 30-year-old adults. Considering MVPA, four days would be necessary to represent a week of measurement among all cohort groups.

**Conclusion:**

Our results show that four and five measurement days are needed to estimate all habitual PA constructs, for children and adults, respectively. Also, among 30-year-old adults, it is important to make efforts towards weekend days measurement.

## Introduction

Physical activity (PA) has positive effect on health and quality of life of individuals and communities [1]. Evidence shows that PA is a protective behavior on major non-communicable diseases, such as coronary heart disease, type II diabetes and cancer [2]. However, global PA prevalence is still low, placing PA promotion as a priority in the public health agenda and, therefore, demonstrating the need of monitoring PA at population levels [3].

Accelerometers (portable motion sensors) have been increasingly adopted in large scale studies, since they provide more accurate physiological and mechanical parameters to estimate PA [4]. In the last decade, the use of accelerometers for this purpose increased significantly [5], due to the capability to quantify duration, frequency and intensity of PA through acceleration signals, movement patterns and magnitude [6]. Also, currently available accelerometers enable large quantity of data storage, a variety of cut-off points for different PA intensities, movement pattern recognition and the possibility of more detailed analyses using the raw data [5].

For a better understanding of the prevalence, levels and impact of PA on health, accurate measures are essential. However, with the increase of accelerometer-based research, the variability among protocols is also rising, since there is no standardized recommendation for data collection. Also, there are different sources of variability, such as accelerometers brands, placements, unit of analysis, minimum number of hours per day and minimum number of measurement days [7,8]. All these decisions will influence final PA estimates at some degree, and therefore must be discussed according to their advantages and disadvantages.

Therefore, the literature regarding the PA monitoring frame presents some evidence that a high percentage of participants are usually excluded from analysis for not being considered as presenting sufficiently valid accelerometer data across different age groups [9]. Furthermore, how representative is the available data when compared to the habitual PA period is not often discussed. Considering that, in reduced sample sizes, differential losses are frequent between subgroups and PA estimates might be biased[10].

Historically, considering that during the first two decades of accelerometer use these measurements have been conducted by waist/hip-worn devices, a decision regarding the minimum number of hours per day was crucial to define valid days of measurement and, consequently, the minimum number of days to estimate habitual physical activity. However, studies using wrist-worn accelerometers usually adopt a 24 hours-protocol, which increases compliance [5,9]. Therefore, the remaining issue is the minimum days of measurement, which is an important decision defining the logistics of the study and representativeness of habitual PA behavior.

The wrist-worn accelerometry literature regarding this issue has not come to a consensus yet, however some studies have analyzed the ideal number of days for accelerometry measurement [11–13]. Among adults, around two or three days of measurement are required depending on the evaluated PA construct [11,13,14]. In addition, Scheers et al.[15] suggest that PA should be measured for two weekend days and three weekdays in order to achieve a common week reliability in adults. Also, some studies recommend the inclusion of weekend days on PA estimates [16–18]. Among children, the widely accepted recommendation seems to be between three and four measurement days [7,12].

In this sense, it is imperative to discuss not only how many days of measurement will be needed to adequately measure PA, but also how many days will be considered valid. Moreover, scientific literature regarding this subject is mostly from high income settings and low- and middle-income countries (LMICs) must be studied due to potential differences in terms of compliance of accelerometer use as well as regarding PA behavior, such as the distribution of PA according to different domains. Therefore, the present study aims to estimate the minimum number of accelerometer measurement days needed to estimate habitual PA among 6-, 18- and 30-year-old participants, belonging to three population-based Brazilian birth cohorts.

## Materials and methods

### Study design and participants

This study is based on data from three birth cohorts studies conducted in Pelotas (a southern city in Brazil) where PA was objectively assessed between 2010 and 2012. All babies delivered in hospital in 1982, 1993 and 2004 were identified during daily visits to the maternity hospitals in the city (more than 99% of deliveries occur in hospitals). In each cohort, less than 1% of participants recruited refused to participate. All three cohorts have been followed up at different time points thereafter. More details regarding the methodology of each cohort have been previously described elsewhere [19–21].

Recent follow-up visits were performed when cohort participants born in 1982, 1993 and 2004 were approximately 30 years, 18 years and 6 years of age, respectively. At these follow-ups, participants were interviewed and clinically examined by the research team. Data on socioeconomic and demographic characteristics, anthropometry, clinical and biochemical measurements were collected. After the clinical examinations, participants were invited to wear an accelerometer.

Approval for the study was obtained from the School of Medicine Ethics Committee of the Federal University of Pelotas and all participants or their legal representatives voluntarily signed written informed consent.

#### Physical activity measurement

PA was assessed by triaxial wrist-worn accelerometers (GENEActiv; ActivInsights, Kimbolton, UK) on the non-dominant wrist. This device has been previously validated and applied in calibration studies among children and adults[22–24]. The GENEActiv Accelerometer measures body movements on three axes: vertical (Y), horizontal right-left (X), and horizontal front-back axis (Z), within an acceleration dynamic range of ± 8*g*, where *g* represents gravitational unit. Sampling frequency was set at 85.7 Hz.

For practical reasons and to increase compliance, participants were invited to wear the accelerometer using a 24 hours protocol (during awakening and sleeping hours) for 4–7 consecutive days, including at least one weekend day. The total amount of monitored days varied according to the day of the clinical visit. Participants who visited the clinic either on Mondays, Tuesdays or Wednesdays were monitored until the following Monday, whereas those who visited the clinic either on Thursdays, Fridays or Saturdays, were monitored until the following Wednesday. Further information regarding the protocol is available elsewhere [25]. After applying the non-wear time criteria, only part of the cohort groups achieved six 24 hours cycles with valid data. All analyses in the present study were restricted to individuals who provided at least six valid days, respecting the valid day criteria of minimum of 16 hours of measurement.

### Data reduction

The device set up and data download was performed in the GENEActiv software. Accelerometer data in binary format were analyzed with R-package GGIR [http://cran.r-project.org] [24]. The detailed signal processing scheme included the following steps: verification of sensor calibration error using local gravity as a reference; detection of sustained abnormally high values (pattern non-compatible with human movement) and non-wear detection. Furthermore, calculation of the vector magnitude of activity-related acceleration Euclidian Norm Minus One (ENMO) was used to summarize three-dimensional raw data (from axes x, y, and z) into a single-dimensional signal vector magnitude. The data were further summarized when calculating the average values per 5-second-epochs (ENMO = ∑ |$\sqrt{x^{2}{+y}^{2}+z^{2}}$ – 1*g*|)[24].

The summary measures used in the present study were (1) overall PA (the average ENMO per day—expressed in m*g*)[24], (2) moderate-to-vigorous PA (MVPA) per day (expressed in minutes) with bouts of 10 minutes, and (3) light PA (LPA) (expressed in minutes). MVPA was defined as ENMO records above 100mg, while bouts criterion was defined as consecutive periods in which participants spent at least 80% of time in MVPA. LPA was considered as activities with acceleration between 50mg and 100mg[22].

### Statistical analyses

Descriptive analyzes comparing characteristics of individuals with six days of data and the remaining participants of the cohorts were performed using ANOVA or its non-parametric equivalent when necessary. Absolute and relative frequencies distribution and respective p values were presented, according to sex (male/female), weight status (underweight, normal-weight, overweight and obese) and asset index. The BMI estimates were based on the World Health Organization (WHO) classification of BMI for adults (1982 cohort) [26], according to the age and sex Z-scores for BMI for children and young adults (1993 and 2004 cohorts)[27]. Asset index was obtained based on a list of assets. These assets were inserted in a principal component analysis to calculate a score and divided into quintiles where the first one indicates the poorest group[28].

Also, description of PA measures (medians of overall PA, MVPA, LPA) between weekdays and weekend days were conducted, and statistical differences were tested with chi-squared and Kruskal-Wallis tests. The reliability for accelerometer minimum days was obtained using the Spearman Brown Formulae[29], which is based on Intraclass Correlation coefficient (ICC) combined with the Spearman Brown Prophecy, applying the ICC of a single day to a different number of days. The reliability provides the proportion of “real” information about a construct of interest, e.g. if a reliability of 0.70 is found, it can be concluded that 70% of the variability in the measurement represented the construct, and 30% represented random variation[30]. All analyses were stratified by age groups (birth cohorts) and performed in the software Stata 12.0. Statistical significance was set at 5%.

## Results

Sample description, as well as comparison between individuals with six measurement days (analytical sample) and the remaining members followed-up on the most recent data collection of each cohort is presented in Table 1. There were no significant differences between the analytical sample and the rest of the cohort in terms of socioeconomic position and BMI groups. The only significant difference observed was between gender among 18-year-old young adults, presenting a higher proportion of females in the analytical sample compared to the remaining cohort participants, which included more males (p = 0.005).

**Table 1:** Sample characteristics of 6-, 18- and 30-year-old individuals that wore the accelerometer for six and less than six measurement days.

	6 years			18 years			30 years		
Variables	< 6 days (n = 2,500)	≥ 6 days (n = 103)		< 6 days (n = 3,090)	≥ 6 days (n = 503)		< 6 days (n = 2,260)	≥ 6 days (n = 452)	
	n (%)	n (%)	p value	n (%)	n (%)	p value	n (%)	n (%)	p value
**Gender**			0.132			0.005			0.410
Male	1,294 (51.8)	47 (45.6)		1,487 (48.1)	276 (54.9)		1,099 (48.6)	210 (46.5)	
Female	1,206 (48.2)	56 (54.4)		1,603 (51.9)	227 (45.1)		1,161 (51.4)	242 (53.5)	
**Asset index (quintiles)**			0.147			0.741			0.349
1º (poorest)	246 (10.1)	10 (9.8)		616 (20.1)	104 (20.7)		514 (24.4)	121 (28.7)	
2º	371 (15.3)	23 (22.6)		615 (20.0)	91 (18.1)		453 (21.5)	86 (20.4)	
3º	574 (23.6)	15 (14.7)		608 (19.8)	109 (21.7)		566 (26.8)	100 (23.7)	
4º	489 (20.1)	22 (21.6)		631 (20.5)	105 (20.9)		195 (9.2)	42 (10.0)	
5º (richest)	750 (30.9)	32 (31.4)		603 (19.6)	93 (18.5)		383 (18.1)	73 (17.3)	
**BMI**			0.060			0.600			0.958
Normal	1,487 (63.9)	70 (72.9)		2,189 (72.3)	368 (73.9)		889 (41.0)	175 (40.3)	
Overweight	434 (18.7)	18 (18.8)		543 (17.9)	80 (16.1)		755 (34.8)	154 (35.5)	
Obesity	405 (17.4)	8 (8.3)		297 (9.8)	50 (10.0)		524 (24.2)	105 (24.2)	

Missing values: Asset index at 6 years(n = 1), 18 years(n = 1) and 30 years(n = 30); BMI at 6 years (n = 7), 18 years (n = 5) and 30 years(n = 18)

**Fig 1:**
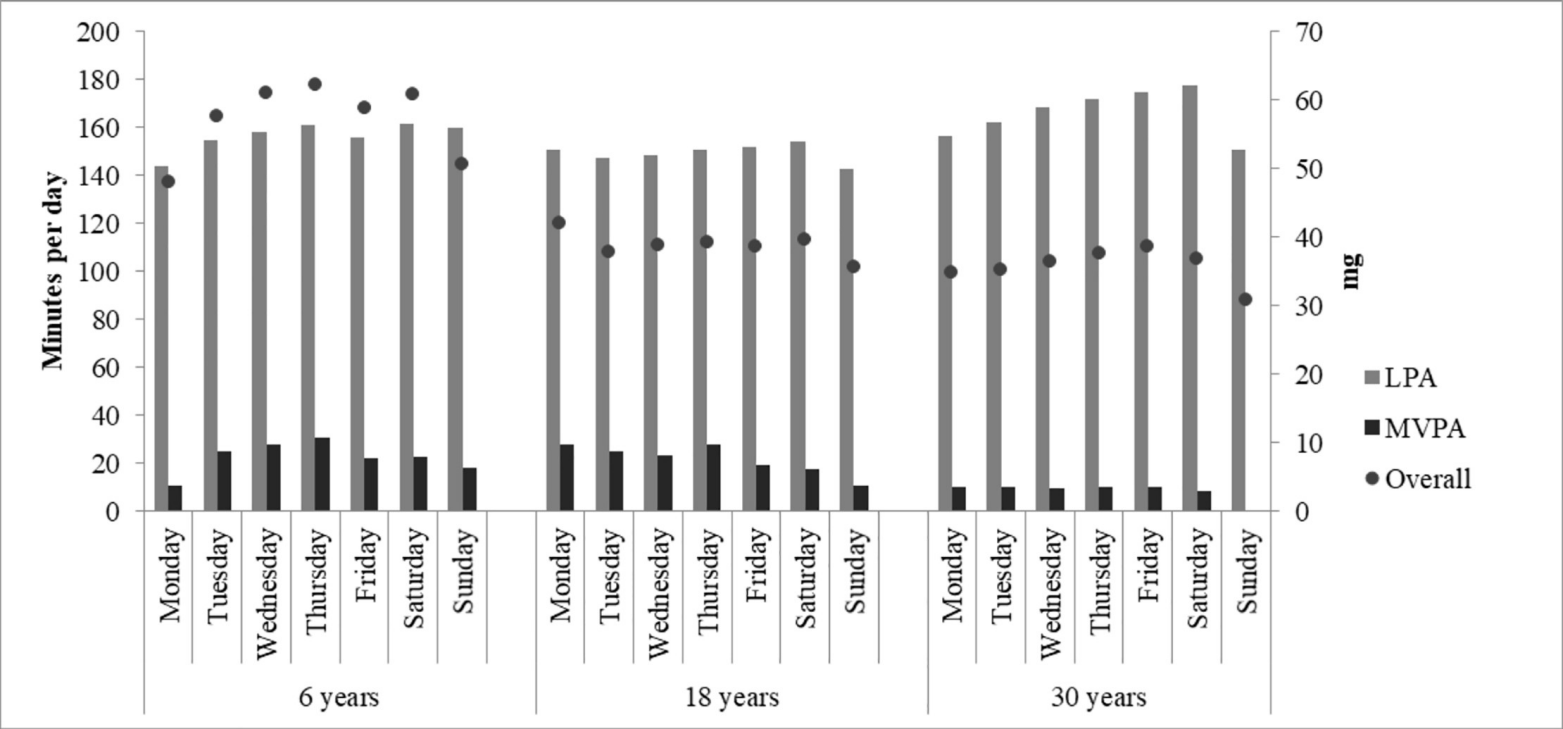
Median of minutes spent in light and moderate-to-vigorous physical activity and overall acceleration (expressed in m*g*) at 6-, 18- and 30-year-old individuals (number of observations in brackets).

**Fig 2:**
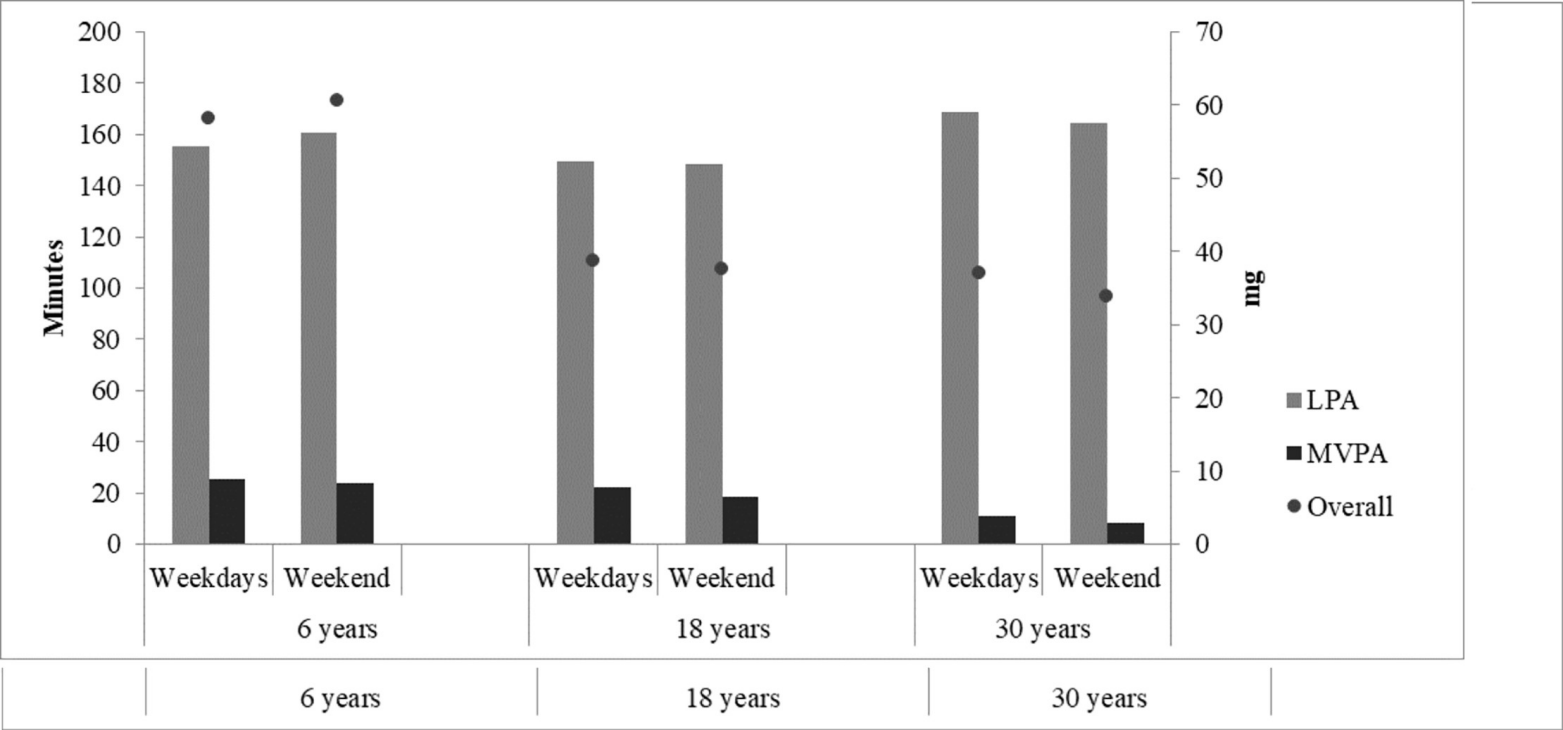
Description of physical activity median spent on light and moderate-to-vigorous physical activity (minutes) and overall acceleration (expressed in m*g*) during weekdays and weekend days among 6-, 18- and 30- year-old individuals.

**Fig 1** presents the median of minutes spent per day in LPA and MVPA, respectively, and the median of overall PA. Among adults (18- and 30- year-old), overall PA, MVPA and LPA median were lower on Sundays compared to the rest of the week (p<0.05 for all estimates in both age groups). MVPA medians on Sunday for the 30- year-old group was 0. Among children, overall PA and LPA were lowest on Mondays (overall PA: p = 0.003; LPA: p = 0.012). Differences between week and weekend days regarding LPA, MVPA and overall PA, were only observed among 30-year-olds. In this cohort group, more MVPA (p = 0.006) and overall PA (p<0.001) were performed on weekdays (**Fig 2**).

**Fig 3:**
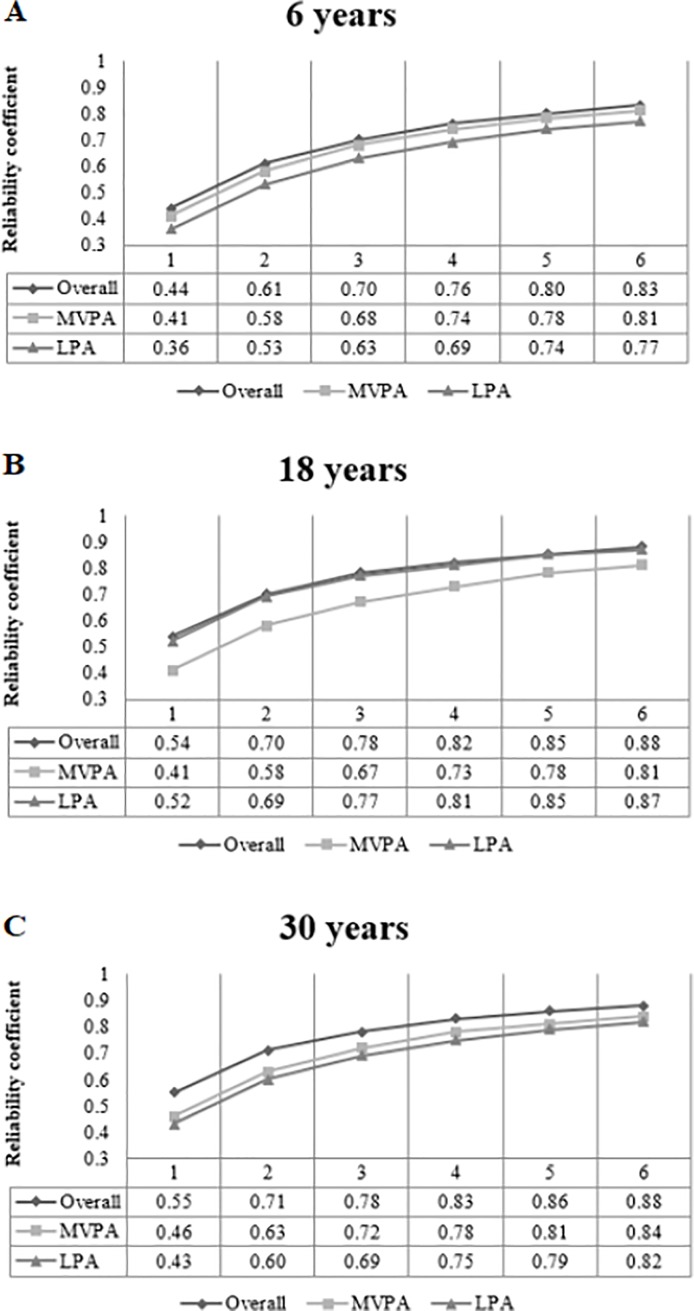
Reliability coefficient of 1 to 6 measurement days on overall, light and moderate-to-vigorous physical activity among 6-year-old children (A), 18-year-old young adults (B) and 30-year-old adults (C).

**Fig 3A, 3B** and **3C** present the reliability coefficient for one to six measurement days. Highest reliability coefficient was observed for overall PA in all three groups. The coefficients regarding overall PA ranged from 0.44 to 0.83 in children and from 0.54 to 0.88 in adults. Also, for overall PA, to achieve a reliability coefficient >0.70, two and three days of measurement were needed in adults and children, respectively. Three and four days of measurements of MVPA was needed to achieve a reliability coefficient >0.70 in 30-year-olds and in 18- and 6-year-olds, respectively. Five days of LPA was necessary to reach a reliability coefficient >0.70 in 6-year-old children, three days for 18-year-old young adults, and four days for 30-years-old adults.

## Discussion

Our results show that, five and four days of measurement are needed to adequately estimate all constructs of PA among children and adults, respectively. Considering MVPA, to represent a week of measurement, three days would be needed for children and four days for adults. Overall PA follows a stable pattern through the week, resulting in a smaller number of days needed to an admissible reliability coefficient, being two days for adults and three for children.

A better interpretation of our results requires the understanding of the main characteristics of the analyzed PA constructs. For instance, overall PA demonstrates the behavior with minimum arbitrariness, including all daily movement, from ordinary daily life activities to physical exercise. Similarly, light PA as treated in the present study, non-bouted, represents low intensity activities without any duration restriction, but excluding sedentary time and MVPA. Lastly, when using 10 minutes bouts, MVPA estimates attempt to represent structured activities performed for longer periods, such as sports, long periods of walking and running (as exercise), etc. Therefore, it is expected to find different reliability results among the outcomes, once they represent distinct constructs of PA.

Opposite to our results, other studies have reported differences regarding weekdays and weekend days among children and adults [11,31,32]. This may be due to our specific sample, which includes only 6, 18 and 30-year-old individuals, not an age range (e.g. 18 to 60-year-old individuals). However, it is important to highlight that reliability is not just an age-related phenomenon, it is also a cultural aspect and observed reliability estimates in the present study does not necessarily translate to other settings.

Most previous studies examining reliability, or other measures to establish the minimum number of measurement days to achieve a desirable reliability coefficient have usually examined different PA intensities such as light, moderate, vigorous or MVPA[11,15]. However, overall PA assessment is also relevant, because it encompasses all PA intensities with lower arbitrariness, therefore illustrating a continuum of PA [14]. Also, considering the issue of lack of comparability among accelerometry literature, assessing overall PA expressed in raw acceleration (m*g)* can facilitate a direct comparison between studies[33].

Regarding children, more days of measurement were necessary to achieve reliability coefficient >0.70, for all three PA measures. This could be due to lack of precision related to small sample size in our youngest group compared to adults and to other studies with children. Addy et.al. [12] found that 3.6 days of measurement would be needed to achieve an ICC of 0.70 among preschool children. Another study, using wrist-worn accelerometers showed a reliability coefficient of 0.8 with two days evaluating adults’ MVPA[11]. This evidence shows that many factors could impact on the decision of how many days should be included in accelerometry data analyses, such as: a) reliability coefficient ideal threshold, b) study population, and c) the PA construct of interest.

Therefore, it is important to consider an accelerometry protocol including at least a week of measurement, in order to guarantee reliable estimates of PA. Evidence from two weeks of adults’ 24 h-accelerometry-assessed data, shows that seven days are necessary to estimate overall PA, and between six and nine days for PA intensities[34]. For children, the literature is not consistent on how many days would be necessary for a reliable estimate of habitual PA, ranging from four to nine days[35]. Also, different protocols are needed depending on the research question. Surveillance studies may be satisfied with only one day of data while studies about seasonality need many days in different periods of the year. Furthermore, the present analyzes are recommended for each data collection *a posteriori*, in order to define the minimum of valid days of measurement in each study, balancing between sample size and reliability estimates[10].

We observed no differences in any of the PA constructs between weekdays and weekend days, except for 30-year-old adults, in which MVPA and overall PA levels were lower on weekend days. Likewise, adults presented lower PA on Sundays in the daily comparison analyzes. This may occur due to the decrease in workload and routine activities on Sunday. The Brazilian legislation determines employers must guarantee the Sunday as holiday for most jobs [36] therefore, many people work from Monday to Saturday. For 18-year-old young adults, the analyzes between weekend and weekdays’ PA did not present significant difference, possibly because Saturday presents similar results than the remaining week, diluting the difference between weekdays and weekend days. Also, children may present different patterns of PA then adults when comparing weekdays and weekend days.

Addressing the difference between weekdays and weekends for adults (30-year-old adults), can be more complex than simply including Saturday or Sunday in the final measure. As shown in our study, MVPA on Sunday was much lower compared to Saturday and weekdays. This finding indicates a potential dissimilar PA pattern for adults on Sundays, which may lead researchers to rethink data analysis and interpretation in this case. The recommendation to include at least one weekend day in the analysis is frequent in the literature [35,37]. However, it seems to be important to discuss if it would be better to include a completely different day, in terms of PA patterns, in the weekly averages, which would have an impact on the weekly estimates; or to treat this day separately in the analysis. These topics might lead to different methodological decisions depending on the research question and the evaluated outcomes.

The present study is based on three large population-based birth cohorts born 11 years apart, with objective measures of PA, providing estimates of the number of days needed to obtain a desirable reliability coefficient on different age groups. Also, to our knowledge, this is the first Brazilian study to evaluate reliability of wrist-worn accelerometry-based PA data.

However, some limitations need to be addressed, such as the decrease in the number of observations when restricting the analyzes to those with six consecutive days, mainly among children. This decrease is due to the use of the non-commercial version of the GENEA accelerometer in the beginning of the data collection. Analyses (not showed here) concluded that such information would not be comparable to the GENEActiv and were not analyzed in the present study. Further explanation on this issue is available in a previous publication[25]. This decrease in sample size is an example of the issue discussed by Migueles, et al. (2017), that elevating valid week criteria provides greater reliability but may also compromise sample size [10].

Also, in the present study we did not exclude sleep periods from the analyses, therefore overall PA represents all body movement in the period. This may impact on our results, specially the comparison between weekdays and weekend days, since sleep duration is increased during weekend days[38]. Lastly, we applied general cut-point thresholds for all age groups since validation/calibration studies of wrist-worn accelerometers are still incipient, which might be considered an important limitation for this area of measurement. In this matter, there is need for more calibration studies and independent validation studies for current available cut-points in order to move towards a consensus[39].

Another issue in accelerometry-based literature is the lack of studies in LMICs, where PA practices differ from high income settings [25,40]. Also, the improvement of measurement tools for physical activity surveillance would enhance the quality and coverage of physical activity data in LMICs [41]. In this sense, regarding the generalizability of our results, it is important to keep in mind that we studied a specific sample, including 6-, 18- and 30-year-old individuals. Although our findings are relevant for LMICs settings, they are not necessarily representative of children or adults in general.

In conclusion, our results show that four and five measurement days are needed to estimate all habitual PA constructs, for children and adults, respectively. Also, among 30-year-old adults, it is important to make efforts towards weekend days measurement. However, final decisions on the research protocol of accelerometer data collection should balance according to each research question, available logistic and potential losses of valid days during the process.

## What does this article add?

Since we found differences between weekdays and weekend days physical activity among adults, it is important to make efforts towards weekend days measurement in order to well represent the physical activity variability across the week. The key finding is that the number of measurement days required to estimate physical activity depends on the age range and the construct of physical activity evaluated, varying between three and five measurement days. When evaluating children’s physical activity, there is a need for more days of measurement when compared to adults, regardless of the construct of physical activity.

## Supporting information

S1 Table Median of minutes spent in light and moderate-to-vigorous physical activity and overall acceleration (expressed in mg) at six, 18- and 30-years old individuals in different days of the week. (DOCX)

S1 Dataset Individual accelerometry and sociodemographic data. (XLSX)
