# Supplementary material for: Number of days required to estimate physical activity constructs objectively measured in different age groups: Findings from three Brazilian (Pelotas) population-based birth cohorts
Source: PLoS One. 2020 Jan 10;15(1):e0216017. doi: 10.1371/journal.pone.0216017 (PMC6953881; doi:10.1371/journal.pone.0216017)
Supplement: S1 Table — (DOCX) [file pone.0216017.s001.docx]

**Suplementary table 1.** Median of minutes spent in light and moderate-to-vigorous physical activity and overall acceleration (expressed in m*g*) at six, 18- and 30-years old individuals in different days of the week

|  | **6 years** | | **18 years** | | **30 years** | |
| --- | --- | --- | --- | --- | --- | --- |
|  | **Median** | **IQ range** | **Median** | **IQ range** | **Median** | **IQ range** |
| **LPA** |  |  |  |  |  |  |
| Monday | 145 | 119 -167 | 148 | 122 - 180 | 147 | 114 - 187 |
| Tuesday | 156 | 134 - 173 | 144 | 111 - 177 | 163 | 125 - 199 |
| Wednesday | 160 | 139 - 176 | 143 | 112 - 181 | 168 | 133 - 204 |
| Thursday | 164 | 145 - 180 | 150 | 118 - 179 | 172 | 136 - 206 |
| Friday | 158 | 138 - 172 | 150 | 115 - 185 | 173 | 138 - 208 |
| Saturday | 158 | 140 - 183 | 149 | 116 - 191 | 172 | 136 - 210 |
| Sunday | 162 | 136 - 180 | 139 | 106 - 174 | 147 | 112 - 183 |
| **MVPA** |  |  |  |  |  |  |
| Monday | 11 | 0 - 30 | 28 | 10 - 67 | 10 | 0 - 39 |
| Tuesday | 25 | 0 - 49 | 25 | 0 - 53 | 10 | 0 - 31 |
| Wednesday | 28 | 9 - 57 | 23 | 0 - 53 | 10 | 0 - 34 |
| Thursday | 30 | 9 - 93 | 28 | 0 - 57 | 10 | 0 - 36 |
| Friday | 22 | 8 - 52 | 20 | 0 - 49 | 10 | 0 - 36 |
| Saturday | 23 | 0 - 56 | 17 | 0 - 51 | 8 | 0 - 25 |
| Sunday | 18 | 8 - 49 | 11 | 0 - 38 | 0 | 0 - 17 |
| **Overall PA** |  |  |  |  |  |  |
| Monday | 45.1 | 32.6 - 54.1 | 40.1 | 29.6 - 53.5 | 33.4 | 24.9 - 42.5 |
| Tuesday | 55.2 | 40.9 - 67.7 | 35.4 | 27.7 - 45.2 | 32.6 | 26.0 - 42.3 |
| Wednesday | 57.6 | 46.4 - 70.7 | 36 | 27.2 - 46.1 | 33.6 | 27.4 - 43.3 |
| Thursday | 62.2 | 47.9 - 74.7 | 36.3 | 28.4 - 48.4 | 35.7 | 28.8 - 43.5 |
| Friday | 57 | 40.9 - 72.5 | 35.5 | 27.4 - 46.5 | 36.3 | 28.8 - 46.0 |
| Saturday | 56.9 | 43.1 - 73.2 | 36.9 | 27.7 - 47.3 | 34.4 | 26.6 - 42.8 |
| Sunday | 58.5 | 43.3 - 74.6 | 31.8 | 25.2 - 41.3 | 28.8 | 22.8 - 37.4 |
